# Neurofilament light chain concentration mediates the association between regional cortical thickness and Parkinson’s disease with excessive daytime sleepiness

**DOI:** 10.3389/fnagi.2025.1645290

**Published:** 2025-08-14

**Authors:** Jieyu Chen, Guoliang Jiang, Yongyun Zhu, Chunyu Liang, Chenxi Liu, Jianzhun Chen, Baiyuan Yang, Xinglong Yang

**Affiliations:** ^1^Department of Neurology, The First Affiliated Hospital of Kunming Medical University, Kunming, China; ^2^Department of Neurosurgery, The First Affiliated Hospital of Kunming Medical University, Kunming, China; ^3^Department of Neurology, Seventh People's Hospital of Chengdu, Sichuan, China

**Keywords:** cortical thickness, mediation analysis, Parkinson’s disease, neurofilament light chain, excessive daytime sleepiness, functional connectivity, neuroimaging biomarkers

## Abstract

**Background:**

Excessive daytime sleepiness (EDS) is a common non-motor symptom in Parkinson’s disease (PD) that negatively impacts quality of life. Although biomarkers of brain structure, function, and neurodegeneration have been studied, their interactions in EDS remain unclear. This study explores the relationship between cortical thickness, functional connectivity (FC), and plasma neurofilament light chain (NfL) levels in PD-EDS.

**Methods:**

36 PD-EDS patients and 100 PD patients without EDS (PD-non-EDS) underwent structural MRI and resting-state FC analysis, with regions of cortical atrophy serving as regions of interest (ROIs). Plasma NfL levels were quantified using high-sensitivity Single Molecule Array (SiMoA™). Mediation analysis was conducted to explore the interplay between NfL levels, neuroimaging markers, and EDS severity, assessed by the Epworth Sleepiness Scale (ESS).

**Results:**

PD-EDS patients exhibited significant cortical thinning in the left supramarginal gyrus (SMG) and right postcentral region (PoCR), along with weakened FC between the left SMG and left PoCR, and between the right PoCR and left inferior frontal gyrus (all *p* < 0.05). Plasma NfL levels were significantly higher in PD-EDS patients than in those without EDS (*p* = 0.004) and mediated the relationship between left SMG thickness and EDS severity.

**Conclusion:**

Plasma NfL levels mediate the association between cortical thinning in the left SMG and EDS severity in PD-EDS, suggesting a link between neurodegenerative processes underlying axonal injury and cortical atrophy in key regions associated with EDS in PD. Our findings suggest that combining neuroimaging markers with plasma NfL levels may provide valuable insights into the mechanisms driving EDS progression in PD.

## 1 Introduction

Excessive daytime sleepiness (EDS) is a common non-motor manifestation of Parkinson’s disease (PD) impacting nearly 50% of patients (Abbott and White, 2005). Characterized by inappropriate drowsiness during wakefulness, EDS impairs cognitive function and quality of life and increases risks such as traffic accidents (Knie et al., 2011). EDS is more frequently observed in advanced stages of PD and has been associated with various non-motor features, such as mood disturbances, autonomic dysfunction, and fatigue (Feng et al., 2021; Maggi et al., 2023). Though its exact mechanisms remain unclear, damage to wake-promoting brain regions and neurotransmitter imbalances—particularly in dopaminergic, cholinergic, and noradrenergic systems—are likely contributors (Liu et al., 2022). Identifying reliable biomarkers and understanding its neurobiological underpinnings is crucial for early intervention and disease management (Siddiqui et al., 2024; Tripathi et al., 2024).

Neuroimaging research has shed light on structural and functional brain changes in PD-EDS patients. Using Voxel-based morphometry (VBM), researchers have detected gray matter disruptions in regions involved in sleep–wake regulation (Kato et al., 2012; Chondrogiorgi et al., 2015; de Schipper et al., 2017). Surface-based morphometry (SBM), which is more sensitive than VBM in detecting subtle structural changes, has shown that cortical folding measurements, especially cortical thickness, better identify PD-related gray matter alterations (Pereira et al., 2012). SBM-based EDS findings include hypertrophy in the putamen and pallidum (Gong et al., 2019), cortical surface expansion in the anterior insula, and subcortical atrophy in the amygdala and putamen (Rosinvil et al., 2024). Research on cortical thickness in PD-EDS is limited, and conflicting findings underscore the need for further investigation of its impact on brain structure. Functional MRI (fMRI) studies have shown abnormal connectivity in cortical and subcortical arousal networks (Wen et al., 2016; Zi et al., 2022; Zheng et al., 2023), especially within the default mode network, where hyperactivity in prefrontal and temporal regions may reflect compensatory or attentional deficits (Ooi et al., 2019; Wang et al., 2020; Zheng et al., 2023).

Neurofilament light chain (NfL), a cytoskeletal protein released during axonal injury, is a well-established biomarker of neuronal damage and degeneration (Gaetani et al., 2019; Sharma et al., 2024). Raised blood NfL concentrations correlate with both motor and non-motor symptoms in PD, offering clinical potential through advancements in ultrasensitive detection techniques like single molecule arrays (Simoa™; Pilotto et al., 2021; Zhu et al., 2021; Yin et al., 2022). Although one study has revealed elevated plasma NfL levels in patients with PD and EDS (Lin et al., 2024), the relationship between NfL levels and specific neuroimaging markers (Preische et al., 2019; Sampedro et al., 2020; Cruz-Gomez et al., 2021; Lee et al., 2022; Clarelli et al., 2024; Yao et al., 2024) has only been explored in patients with cognitive dysfunction (Mielke et al., 2019). Until now, no prior studies have assessed the tripartite relationship among plasma NfL levels, neuroimaging markers, and EDS severity in PD, representing a pivotal knowledge gap.

This study is the first to integrate structural and functional neuroimaging to investigate the neural mechanisms underlying EDS in PD. Using FreeSurfer, we analyzed cortical thickness alterations in key brain regions and examined functional connectivity (FC) patterns across brain networks. Furthermore, we assessed the interrelationships among cortical thickness, FC, and plasma NfL levels, and their associations with EDS severity. Finally, mediation analyses were conducted to determine whether NfL mediates the relationships between cortical thinning, FC alterations, and EDS, providing insights into potential neurodegenerative pathways.

## 2 Methods and materials

### 2.1 Participants

136 PD patients were recruited from the Neurology Department and outpatient clinics at the First Affiliated Hospital of Kunming Medical University between June 2021 and December 2024. The diagnosis was established based on the 2015 criteria of the International Parkinson’s and Movement Disorders Association (Postuma et al., 2015).

Sleep-related symptoms were assessed through face-to-face interviews using the Epworth Sleepiness Scale (ESS), a validated tool endorsed by the Movement Disorder Society (MDS) for evaluating daytime sleepiness (Högl et al., 2010). Participants were classified into two groups: PD-EDS (*n* = 36), with an ESS score ≥ 10, and PD-non-EDS (*n* = 100), defined by an ESS score ≤ 9 (Amara et al., 2017). Additionally, age- and sex-matched healthy controls without chronic illnesses were included. The exclusion criteria encompassed: (1) atypical Parkinsonism or secondary PD due to other identified conditions; (2) intracranial organic pathologies like tumors, hematomas, or cerebral infarction; (3) a history of traumatic brain injury or prior intracranial surgery; (4) use of medications affecting sleep, including hypnotics; (5) MRI contraindications; and (6) left-handedness.

Ethical approval was granted by the Ethics Committee of the First Affiliated Hospital of Kunming Medical University (2019-L-46), and the study adhered to the principles of the Declaration of Helsinki. Written informed consent was obtained from all participants, allowing the use of anonymized clinical data for research and publication.

### 2.2 Clinical and neuropsychological measurements

Baseline participant data were extracted from electronic medical records and personal interviews. For PD patients, demographic and clinical details, including age, sex, education level, dopamine receptor agonist usage, and levodopa-equivalent daily dose (LEDD), were recorded. Clinical assessments were performed while patients were in the ‘on’ state. Motor function was evaluated using the Hoehn and Yahr (HY) scale and the Unified PD Rating Scale Part III (Goetz et al., 2008). Depressive and anxiety symptoms were assessed via the Hamilton Depression (HAMD) and Anxiety (HAMA) Scales, respectively (Hamilton, 1959; Hamilton, 1960). Cognitive performance was measured using the Mini-Mental State Examination (MMSE). Rapid eye movement sleep behavior disorder (RBD) was screened using the RBD Screening Questionnaire, with scores exceeding 5 indicating a high probability of RBD (Stiasny-Kolster et al., 2007). To classify PD phenotypes, tremor-dominant and postural instability and gait difficulty (PIGD) scores were calculated based on specific MDS-Unified PD Rating Scale (UPDRS) items (Stebbins et al., 2013). Patients were categorized as tremor-dominant if the ratio of the mean UPDRS tremor score (8 items) to the mean UPDRS PIGD score (5 items) was ≥ 1.15, whereas those with PIGD-dominant PD had a ratio of ≤ 0.90.

### 2.3 Plasma NfL

Upon enrollment, 5 mL of venous blood was drawn into ethylenediaminetetraacetic acid (EDTA) tubes and processed within an hour. Following centrifugation (2,500 × g, 10 min), plasma samples were preserved at −80°C for later analysis. NfL concentrations were quantified using the Simoa NF-light® kit (Quanterix, MA, USA) on a Simoa HD-1 Analyzer, adhering to the manufacturer’s instructions. Each sample was thawed a single time, with automatic four-fold dilution performed by the device. The coefficient of variation for duplicates was 4.2%. Quality control samples (high/low NfL concentrations) were included, all within the expected range. Blinded research assistants conducted the assays to minimize bias.

### 2.4 Image acquisition and preprocessing

A 3.0 T whole-body scanner (Discovery 750w, GE Healthcare, USA) was used for MRI acquisition at the Imaging Department of the First Affiliated Hospital of Kunming Medical University. Standard head coils were utilized for both signal transmission and reception. Participants were instructed to stay relaxed, minimize cognitive activity, and remain awake during the procedure. The imaging protocol incorporated routine sequences, including resting-state fMRI (RS-fMRI) and 3D T1-weighted imaging (3D-T1WI). The 3D-T1WI scans were obtained with the following parameters: voxel size, 1 × 1 × 1 mm; repetition time, 8.2 ms; echo time, 3.2 ms; turn angle, 12; inversion time, 450 ms; matrix, 256 × 256; field of view, 256 × 256 mm; and slice thickness. The RS-fMRI parameters included: 36 slices; slice thickness, 3 mm; no gap; voxel size, 3.5 × 3.5 × 4 mm; volume, 240; repetition time, 2,000 ms; echo time, 30 ms; turn angle, 90°; field-of-view, 224 mm; and matrix, 64 × 64.

Surface-based morphometric analysis was conducted using FreeSurfer 6.0.0. Initially, the NIfTI format was generated from 3D-T1 DICOM images using MRIcron software. The converted data were then processed automatically in FreeSurfer within a Linux Ubuntu environment, which involved motion correction, non-brain tissue removal (e.g., skull extraction), transformation into the Talairach space, subcortical structure segmentation, and gray matter normalization. Additional processing steps involved delineating gray matter boundaries, applying topological adjustments, performing surface deformation, and registering the data to a spherical template. Cortical thickness, measured as the distance from the gray-white matter boundary to the pial surface, was computed for each brain region using a Gaussian smoothing kernel (full-width half-maximum [FWHM] = 10 mm). Finally, all reconstructed datasets were visually examined to evaluate the precision of registration, skull stripping, segmentation, and cortical surface reconstruction.

RS-fMRI data processing was performed using Data Processing and Analysis of Brain Imaging (version 4.5), incorporating Statistical Parametric Mapping (SPM12) and MATLAB 2022b. To minimize artifacts from scanner calibration and subject adaptation, the first 10 time points of each fMRI scan were discarded. The remaining images underwent slice-timing correction with the middle slice as a reference, followed by realignment to compensate for head motion. Participants exhibiting head displacement exceeding 2 mm or rotational movement beyond 2° were excluded, resulting in the removal of 24 subjects (Table 1). T1-weighted anatomical images were co-registered to the mean functional image using a rigid-body transformation and segmented into gray matter, white matter, and cerebrospinal fluid via the DARTEL template. Functional scans were normalized to Montreal Neurological Institute (MNI) space, resampled to 3 × 3 × 3 mm^3^ voxels, and smoothed with a 6 mm FWHM Gaussian kernel. To mitigate noise, linear detrending and temporal bandpass filtering (0.01–0.08 Hz) were applied, while nuisance signals from white matter, cerebrospinal fluid, and Friston-24 head motion parameters (including historical and squared terms) were regressed out.

**Table 1:** Baseline comparison between included and excluded PD patient.

Characteristic	PD-Included (*n* = 112)	PD-Excluded (*n* = 24)	*p* value
Age, years	64.5 (57, 71)	70 (60.5, 73.75)	0.09
Male, *n*,%	61 (54.5%)	11 (45.8%)	0.442
Education (years)	9 (6, 12)	6 (6, 12)	0.308
H-Y grade	2 (1, 3)	2 (1, 3)	0.228
UPDRS-III	25 (16.25, 41)	37.5 (18, 45)	0.233
HAMD	6.5 (3, 16)	5 (2, 18.75)	0.864
HAMA	5 (1, 18.75)	10 (1, 20.5)	0.53
MMSE	27 (24, 29)	26 (20.5, 28.75)	0.202
Selegiline (mg), *n*, %	13 (11.6%)	3 (12.5%)	0.9
Pramipexole (mg)	3 (1, 5)	4 (0.625, 5)	0.875
PSQI	10 (6, 13)	10 (5.25, 13.75)	0.975
LEDD (mg)	187.5 (337.5, 468.75)	362.5 (198.12, 487.5)	0.742
Subtype of PD (TD), *n*,%	63 (56.3%)	10 (41.7%)	0.495
Subtype of PD (PIGD), *n*,%	43 (38.4%)	13 (54.2%)	0.154
RBD, *n*,%	34 (30.4%)	9 (37.5%)	0.054
ESS	5 (2, 10)	2 (5.7.75)	0.59

Values are *n* (%), median (interquartile range) or mean ± SD, unless otherwise noted HAMA, Hamilton Anxiety Scale; HAMD, Hamilton Depression Scale; HC, Healthy controls; LEDD, levodopa-equivalent daily doses; MMSE, Mini-Mental State Examination; NA, not applicable; RBD, rapid eye movement sleep behavioral disorder; UPDRS, Unified Parkinson’s Disease Rating Scale; PD-EDS, Parkinson’s disease with excessive daytime sleepiness; PD-nEDS, Parkinson’s disease without excessive daytime sleepiness, MOCA, Montreal Cognitive Assessment; ESS, Epworth Sleepiness Scale, PSQI, Pittsburgh Sleep Quality Index.

### 2.5 Statistical analysis

Statistical analyses were conducted using SPSS version 27.0 (IBM Corp., Armonk, NY, USA). Categorical data were presented as proportions and assessed via the chi-square test. For normally distributed continuous variables, results were reported as mean±standard deviation and compared using two-sample *t*-tests or analysis of variance. Skewed continuous data were expressed as medians with interquartile ranges and analyzed using the Mann–Whitney U test or Kruskal–Wallis test.

**Figure 1:**
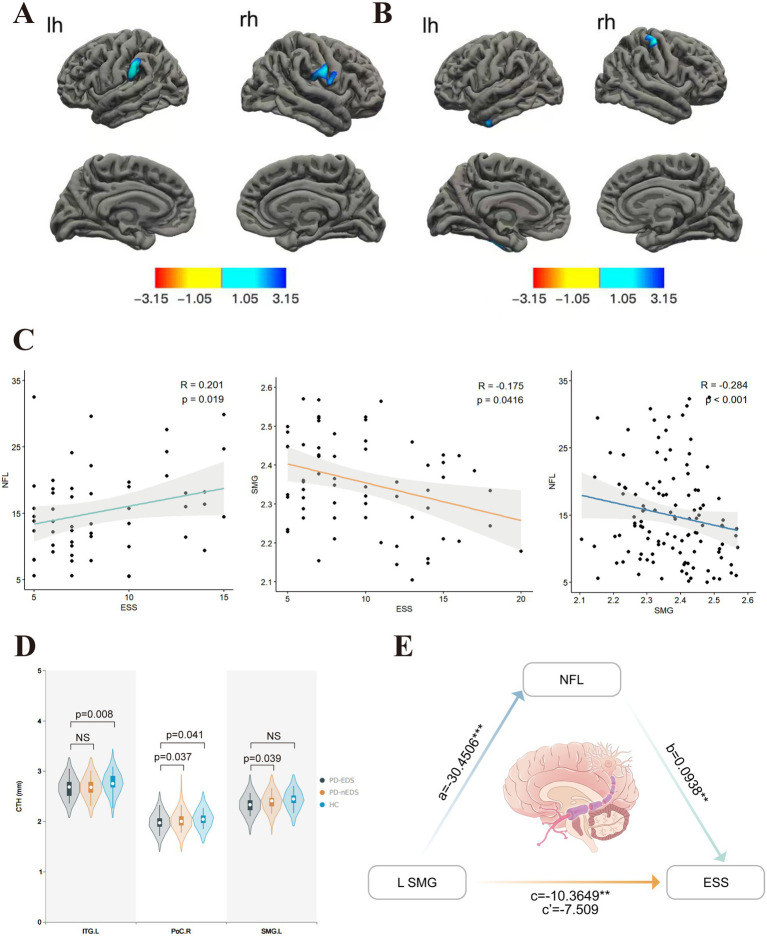
Comparison of cortical thickness in groups covariables are gender, age, education, H-Y. **(A)** In contrast with those without EDS, PD patients with EDS showed lower cortical thickness in SMG. L, PoC.R. **(B)** Compared to HC, PD-EDS patients showed the lower cortical thickness in PoC.R, ITG.L. **(C)** The ESS score was positively associated with plasma NfL concentration. In contrast, it was negatively associated with left SMG thickness, which, in turn, showed a negative association with plasma NfL concentration. **(D)** Cortical thickness values of different clusters between groups. **(E)** Plasma NfL concentration mediated the relationship between left SMG thickness and ESS. The indirect effect of plasma NfL on this relationship was significant, as represented by the paths a and b, which together imply a mediated effect. The direct effect of left SMG thickness on ESS, represented by c’, was not significant after accounting for plasma NfL. NfL, neurofilament light; SMG, supramarginal gyrus; ESS, Epworth Sleepiness Scale; PoC,postcentral gyrus; ITG, inferior temporal gyrus; L, left hemisphere; R, right hemisphere. The red-blue color bar on the figure shows the logarithmic scale of the *p* value (−log10). Red is positive, blue is negative.

Cortical thickness analysis was performed using FreeSurfer’s mri_glmfit function to conduct vertex-wise comparisons based on a general linear model (GLM), adjusting for age, sex, education, and HY status as covariates. Multiple comparisons were corrected using a precached cluster-wise Monte Carlo simulation with 10,000 permutations, identifying significant clusters at a cluster-level threshold of *p* < 0.05 (initial vertex-level threshold *p* < 0.01). Subsequently, mean cortical thickness values were extracted from significant clusters, and correlation analyses were conducted using SPSS version 27.0 (IBM Corp., Armonk, NY, USA), adjusting for the same covariates (Figure 1D). Significance was set at *p* < 0.05.

To investigate cortical thickness differences across groups, corresponding MNI coordinates were extracted and defined as regions of interest (ROIs) with a 10-mm radius. The FC between each ROI and whole-brain voxels was examined using the CONN toolbox in SPM12. The mean BOLD time series was computed for all voxels within each ROI. Bivariate correlation analyses were performed to assess linear associations between the BOLD signals of each ROI pair, followed by Fisher’s z-transformation. Second-level analysis was applied to individual seed-to-voxel maps. All statistical tests were two-tailed, and multiple comparison corrections were implemented using the Gaussian random field method. Results were considered statistically significant at a voxel-level threshold of *p* < 0.001 and a cluster-level threshold of *p* < 0.05. FC differences between the PD-EDS and PD-non-EDS groups were extracted, and Spearman’s correlation was used to assess their relationship with ESS scores.

## 3 Results

### 3.1 Demographic and neuropsychometric characteristics

Baseline demographic characteristics are presented in Table 2. No significant differences were observed in sex, age, or education level between healthy controls and PD patients, regardless of EDS status. Likewise, LEDD, RBD scores, and motor subtypes remained comparable between the PD-EDS and PD-non-EDS groups. However, individuals with PD-EDS exhibited lower MMSE scores, higher PSQI, HAMD, and HAMA scores, as well as elevated plasma NfL levels, compared to their non-EDS counterparts.

**Table 2:** Clinical and demographic characteristics of study participants.

Characteristic	PD-EDS (*n* = 36)	PD-nEDS (*n* = 100)	HC (*n* = 32)	*p* value
Age, years	67.5 (61.25, 74.00)	64 (57, 71)	60 (50, 70)	0.111
Male, *n*,%	24 (66%)	48 (48%)	11 (34%)	0.054
Education (years)	9 (6, 12)	9 (6, 12)	8.5 (8.5, 12)	0.305
H-Y grade	2 (1, 3)	2 (1, 3)	NA	0.09
UPDRS-III	35 (16.75, 45.25)	25 (17, 39)	NA	0.202
HAMD	6.25 (15.5, 25)	2 (5, 11)	NA	<0.001
HAMA	6 (18, 22)	1 (4, 13.75)	NA	<0.001
MMSE	25 (20, 27.75)	28 (25.00, 29.00)	NA	0.002
Selegiline (mg), *n*, %	6 (17%)	10 (10%)	NA	0.287
Pramipexole (mg)	0.375 (0, 0.65)	0 (0,0.46)	NA	0.538
PSQI	11.50 (8.00, 13.00)	9 (5,13)	NA	0.032
LEDD(mg)	325 (200, 443.75)	350 (175, 484.375)	NA	0.57
Subtype of PD (TD), *n*,%	16 (44.4%)	57 (57%)	NA	0.19
Subtype of PD (PIGD), *n*,%	18 (50%)	38 (38%)	NA	0.21
RBD, *n*,%	16 (44.4%)	27 (27.0%)	NA	0.054
ESS	13.50 (10.25, 15.75)	4 (2, 5.75)	NA	<0.001
NFL	18.11 (11.52, 30.58)	13.24 (8.67, 18.54)	11.49 (9.84, 15.08)	0.004

Values are *n* (%), median (interquartile range) or mean ± SD, unless otherwise noted. HAMA, Hamilton Anxiety Scale; HAMD, Hamilton Depression Scale; HC, Healthy controls; LEDD, levodopa-equivalent daily doses; MMSE, Mini-Mental State Examination; NA, not applicable; RBD, rapid eye movement sleep behavioral disorder; UPDRS, Unified Parkinson’s Disease Rating Scale; PD-EDS, Parkinson’s disease with excessive daytime sleepiness; PD-nEDS, Parkinson’s disease without excessive daytime sleepiness, MOCA, Montreal Cognitive Assessment; ESS, Epworth Sleepiness Scale, NFL, neurofilament light chain; PSQI, Pittsburgh Sleep Quality Index.

### 3.2 Alteration in cortical thickness among patients with PD-EDS

Compared to non-EDS patients, individuals with PD-EDS showed reduced cortical thickness in the left supramarginal gyrus (SMG) and postcentral gyrus (PoCR; Table 3, Figure 1A). Similarly, relative to healthy controls, they exhibited cortical thinning in the left inferior temporal gyrus (IFG) and right PoCR (Table 3, Figure 1B).

**Table 3:** Comparison of cortical thickness between groups.

Study group	Brain area	MNI coordinates			Cluster size	Vertex	*p* value
		X	Y	Z			
HC>PD-EDS	Temporal_Inf_L	−43.6	−15.6	−32	464.24	733	0.008
	Postcentral_R	52.5	−18.4	52.4	365.24	783	0.041
PD-nEDS>PD-EDS	SupraMarginal_L	−60.8	−33.1	31.3	398.22	829	0.039
	Postcentral_R	63.6	−9.2	24.7	409.17	982	0.037

PD-EDS, Parkinson’s disease with excessive daytime sleepiness; HC, healthy controls; PD-nEDS, Parkinson’s disease without excessive daytime sleepiness; MNI, Montreal Neurological Institute; SMG, supramarginal gyrus; PoC,postcentral gyrus; ITG, inferior temporal gyrus; L, left hemisphere; R, right hemisphere.

### 3.3 Variations in FC between PD-EDS and PD-non-EDS groups

**Figure 2:**
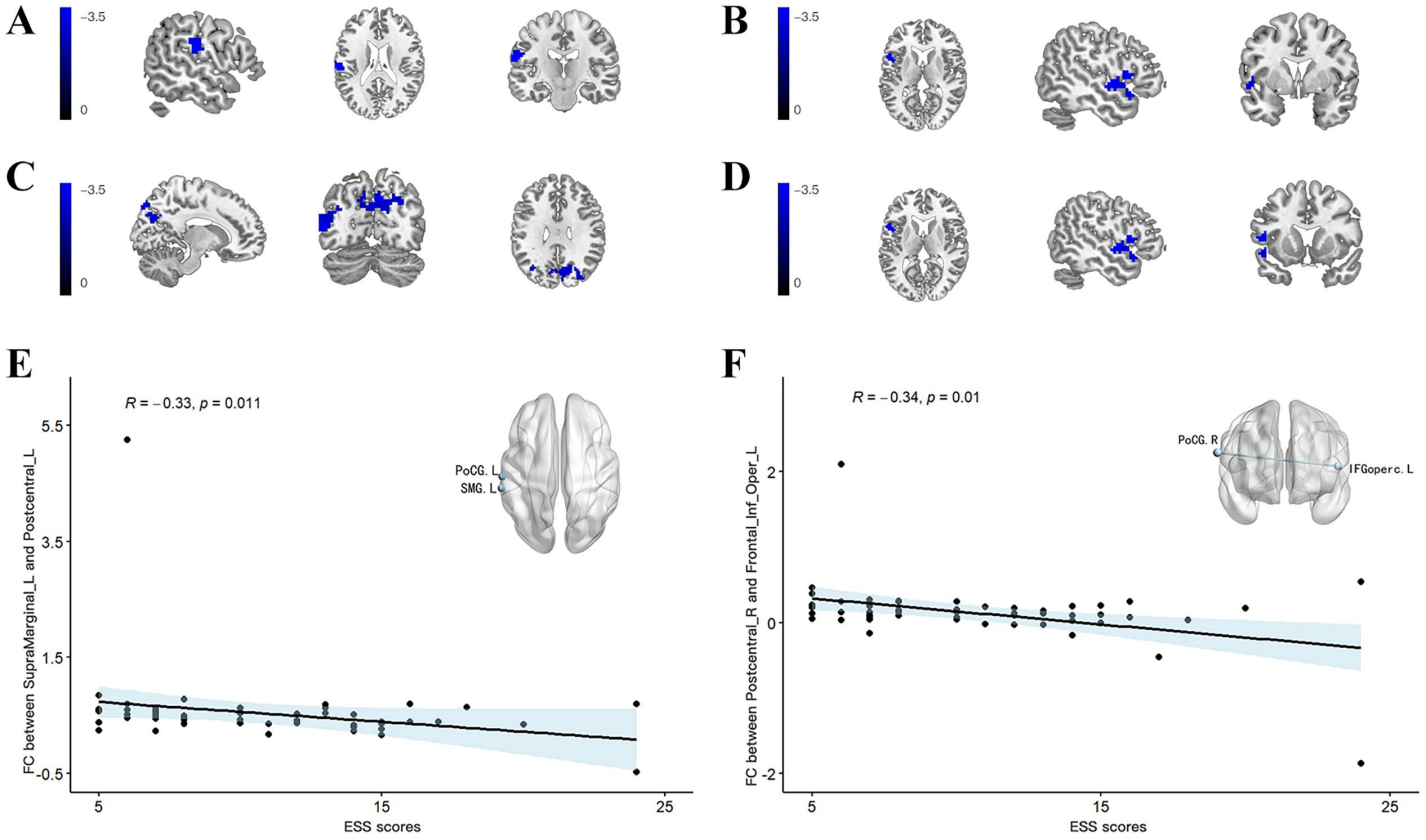
**(A)** Using the SMG_L as the seed region, the PD-EDS group exhibited significantly lower FC compared to PD-non-EDS patients. **(B)** Using the PoC_R as the seed region, the PD-EDS group showed reduced FC relative to PD-non-EDS patients **(C,D)** Using the PoC_R as the seed region, the PD-EDS group showed markedly reduced FC relative to HC group. Regions with weaker connectivity are depicted in blue (T values). **(E,F)** In PD patients, FC between the SMG_L and PoC_L, as well as between the PoC_R and the IFGoperc_R, was negatively correlated with ESS scores.

Using the left SMG as the ROI, patients with PD-EDS exhibited reduced FC with the left PoCR compared to patients without EDS (Table 4, Figure 2A). Similarly, selecting the right PoCR as the ROI revealed decreased FC with the left IFG operc in PD-EDS patients (Table 4, Figure 2B). Using the PoC_R as the seed region, the PD-EDS group showed markedly reduced FC relative to HC group(Table 4, Figures 2C,D).

**Table 4:** Intergroup comparison of functional connectivity in different brain regions.

ROI[MNI coordinates (X, Y, Z)]	Brain regions	MNI coordinates			Vertices	*T* value
		X	Y	Z		
FC difference when PD-EDS minus HC						
Postcentral_R	Temporal_Mid_L	−51	−72	6	133	−4.2218
(52.5,-18.4,52.4)	Cuneus_R	6	−81	36	317	−4.1338
Postcentral_R (63.6,−9.2,24.7)	Paracentral_Lobule_L	-9	−36	63	124	−4.7956
FC difference when PD-EDS minus PD-nEDS						
SupraMarginal_L (−60.8,-33.1,31.3)	Postcentral_L	−60	−21	27	83	−3.744
Postcentral_R (63.6,-9.2,24.7)	Frontal_Inf_Oper_L	−51	12	12	98	−3.6971

ROI, regions of interest; PD-EDS, Parkinson’s disease with excessive daytime sleepiness; PD-nEDS, Parkinson’s disease without excessive daytime sleepiness; SMG,supramarginal gyrus; PoC, postcentral gyrus; IFGoperc, inferior frontal gyrus, pars opercularis; MTG, Middle Temporal Gyrus; CUN, Cuneus; PCL, Paracentral Lobule; L, left hemisphere; R, right hemisphere.

### 3.4 Clinical correlation analysis

Cortical thickness in the left SMG was inversely correlated with both ESS scores and NfL levels (r = −0.175, *p* = 0.0416; r = −0.284, *p* < 0.001, respectively) (Figure 1C). A significant positive correlation was identified between ESS and NfL levels (r = 0.201, *p* = 0.019) (Figure 1C). In PD patients, FC values between the left SMG and left PoCR, as well as between the right PoCR and right IFG operc, showed a negative association with ESS scores (r = −0.33, *p* = 0.011; r = −0.34, *p* = 0.01, respectively). Figure 1D shows the comparison of extracted cortical thickness values among different clusters in each group.

### 3.5 Mediators of plasma NFL

Mediation analysis results indicated that left SMG thickness is indirectly related to ESS through its relationship with plasma NfL concentration (Figure 1E, Table 5). Specifically, a thinner left SMG was linked to higher plasma NfL levels (a = −30.4506, *p* = 0.0005), and a higher plasma NfL level was connected with worse ESS scores (b = 0.0938, *p* = 0.0127). The bias-corrected 95% confidence interval (CI), computed from 10,000 bootstrap samples, confirmed a significant indirect effect of plasma NfL (ab = −2.856) distinct from zero (95% CI = −6.3372 to −0.6079) (Table 5). This suggests that plasma NfL partially mediates the relationship between left SMG thickness and EDS severity, accounting for approximately 27.6% of the total effect. After adjusting for plasma NfL, the direct effect of left SMG thickness on ESS was no longer statistically significant (c′ = −7.509, 95% CI = −15.0905–0.0725, *p* = 0.0522), indicating that the observed association between left SMG thickness and ESS could be partially explained by plasma NfL levels.

**Table 5:** Mediation analysis of the indirect effect of SMG on ESS through NfL.

Effect Path	Unstandardized Coefficient	Standard Error	*t*-value	*p*-value	95% CI (LLCI, ULCI)	Standardized Coefficient (β)
SMG → NfL (a)	−30.4506	8.52	−3.57	0.0005	[−47.30, −13.60]	−0.295
NfL → ESS (b)	0.0938	0.037	2.53	0.0127	[0.0203, 0.1673]	0.218
SMG → ESS (Total effect, c)	−10.3649	3.74	−2.78	0.0063	[−17.75, −2.98]	−0.233
SMG → ESS (Direct effect, c′)	−7.509	3.83	−1.96	0.0522	[−15.09, 0.07]	−0.169
Indirect effect (a × b)	−2.8562	1.51	—	—	[−6.47, −0.58]	−0.064

## 4 Discussion

This study is, to our knowledge, the first to assess how cortical thickness, FC, and plasma NfL levels interact in PD-EDS. Relative to PD patients without EDS, individuals with PD-EDS showed elevated NfL levels, thinner cortices in the left SMG and right PoCR, and weakened FC between the left SMG and left PoCR, as well as between the right PoCR and left IFG operc. Further mediation analysis indicated that plasma NfL was a stronger mediator of the connection between structural brain changes and the severity of EDS in PD. Moreover, the association between cortical atrophy in the left SMG and elevated NfL concentrations may help differentiate PD-EDS from PD-non-EDS, providing a novel perspective on the underlying mechanisms of PD-EDS.

Our study provided the first structural MRI evidence of alterations in the left SMG and right PoCR in patients with PD-EDS. Both regions, located in the parietal lobe, are integral to sensory integration and cognitive processing (Vandenberghe et al., 2012). The PoCR, as part of the primary somatosensory cortex, is essential for tactile perception, spatial awareness, and sensorimotor coordination, whereas the SMG, within the inferior parietal lobule, integrates multisensory information to support higher cognitive functions, including attention and social cognition. Given that PD-EDS is closely tied to cognitive impairment, it is plausible that the atrophy of the parietal cortex, a key hub for attention and sensory processing, contributes to its pathogenesis. Similarly, significant cortical thinning in the medial and dorsolateral prefrontal cortices and inferior parietal lobules has been reported in narcolepsy patients with cataplexy, affecting executive attention and working memory (Joo et al., 2011). Additionally, obstructive sleep apnea is associated with a higher likelihood of developing PD-EDS (Jeon and Oh, 2023), with cortical thinning observed in the left parietal, frontal, and temporal lobes, which is negatively correlated with ESS score (Li et al., 2023). Thus, these findings suggest that cortical thinning in specific regions may be associated with attention deficit and memory impairment in sleep disorders, including PD-EDS.

Cortical atrophy in key parietal regions may disrupt network connectivity and contribute to EDS in PD. Specifically, reduced FC between the left SMG and PoCG may impair sensory integration and attentional regulation. As components of the somatosensory network, the PoCG (S1) and SMG (S2) are involved in processing tactile input and coordinating sensorimotor functions (Potok et al., 2019). Their weakened interaction may reduce arousal-related signaling, thereby increasing vulnerability to EDS (Hitchcott et al., 2019). Furthermore, weakened FC was detected between the right PoCR and left IFG operc. As a subregion of the left inferior frontal gyrus, the IFG operc plays a key role in information processing during audiovisual perceptual decision-making (Li et al., 2020). These findings align with evidence linking frontal cortex dysfunction to PD-EDS (Wen et al., 2016; Ooi et al., 2019; Zi et al., 2022). The impaired white matter tract integrity between the frontal and parietal lobes in patients with PD-EDS further supports the neural downregulation mechanisms underlying EDS (Chondrogiorgi et al., 2015). Reduced FC between these key brain regions may contribute to deficits in sensory processing, attention, and regulation of arousal. Moreover, the weaker connectivity between these brain regions was correlated with higher ESS scores, indicating greater EDS severity. Disruptions in the FC within the left parietal cortex may serve as targets for neuromodulation interventions such as repetitive transcranial magnetic stimulation. Further investigation is necessary to determine how effectively these approaches modulate the arousal and attentional networks in PD-EDS.

NfL is a key cytoskeletal protein in neurons and a well-established biomarker of axonal damage (Petzold, 2005). Our findings indicate that plasma NfL levels are elevated in PD-EDS compared to PD-non-EDS, aligning with previous studies (Lin et al., 2024). Mediation analysis suggested that reduced SMG thickness may exacerbate EDS severity by driving NfL elevation, implying that neurodegeneration-induced cortical thinning promotes axonal injury biomarker release, which subsequently worsens EDS. Previous studies have demonstrated that serum NfL is related to posterior cortical atrophy in early PD, particularly in the parietotemporo-occipital regions, and is a marker of non-dopaminergic neurodegeneration linked to cognitive decline and PD progression (Sampedro et al., 2020). Elevated NfL concentrations have been shown to mediate the relationship between cortical atrophy and cognitive decline in multiple sclerosis patients (Cruz-Gomez et al., 2021). In Alzheimer’s disease, a bidirectional relationship exists, in which higher NfL predicts faster cortical thinning, whereas reduced cortical thickness accelerates NfL elevation, reflecting ongoing axonal degeneration (Mattsson et al., 2019). Combining NfL level with MRI-based cortical thickness measurement enhances the assessment of neuroaxonal injury. However, the exact relationship between EDS-related cortical thinning and increased NfL levels remains unclear. One plausible mechanism is that low perfusion and reduced blood flow may contribute to cortical thinning by causing metabolic dysfunction and oxidative stress, leading to neuronal shrinkage and cortical atrophy. This thinning, in turn, could exacerbate neurodegeneration and worsen EDS severity (Morrison and Hof, 1997). Consistent with our findings, attention deficits and reduced blood flow in the left parietal cortex have been associated with PD-EDS (Matsui et al., 2006).

Our study explored the contributors to EDS in individuals with PD. Consistent with previous research, EDS was strongly associated with non-motor symptoms, particularly cognitive impairments, anxiety, depression, and poor nighttime sleep quality (Kurtis et al., 2013; Zhu et al., 2016; Feng et al., 2021; Maggi et al., 2023). However, in contrast to previous studies that linked EDS to age, disease duration, and PIGD phenotype, there were no significant associations with these variables in our cohort. Notably, mediation analysis revealed that the indirect effect of cortical thinning on EDS through NfL was reduced and became non-significant after controlling for MMSE scores, suggesting that cognitive dysfunction may partially account for this pathway. These findings highlight the predominant influence of non-motor symptoms—especially cognitive impairment—on EDS, relative to disease duration and motor phenotype. Further research with detailed neuropsychological assessments and rigorous control of confounders is required to clarify these relationships.

Despite these important findings, this study had several limitations. First, although ESS is a commonly employed scale for evaluating EDS, it remains a subjective measure. Polysomnography and other objective tests enable a more comprehensive evaluation. However, these tests are resource-intensive and impractical for large-scale studies, making ESS a feasible alternative for screening EDS in clinical and research settings. Second, the EDS group had a relatively small sample size, especially after we excluded participants with excessive head motion, which may have affected statistical power. Validating these findings necessitates larger, multicenter studies. Finally, the cross-sectional design limits causal inferences between NfL, cortical thinning, and EDS severity in patients with PD. To gain a better understanding of PD-EDS mechanisms, longitudinal studies are essential in determining whether elevated NfL levels precede cortical thinning or result from neurodegeneration.

Collectively, our results indicate that elevated plasma NfL partially mediated the interaction between cortical thinning in the left SMG thickness and the severity of EDS, possibly reflecting the crucial role of neurodegeneration in linking cortical atrophy in this region to EDS. Cortical thinning may impair FC and exacerbate EDS in patients with PD. By integrating cortical thickness, FC, and NfL, our study enhances the comprehension of the potential mechanisms of PD-EDS as well as provides insights into the clinical implications and possible therapeutic targets.

## Data Availability

The original contributions presented in the study are included in the article/Supplementary material, further inquiries can be directed to the corresponding authors.
