# Supplementary figures and images for: Neurofilament light chain concentration mediates the association between regional cortical thickness and Parkinson’s disease with excessive daytime sleepiness
Source: Front Aging Neurosci. 2025 Aug 14;17:1645290. doi: 10.3389/fnagi.2025.1645290 (PMC12391049; doi:10.3389/fnagi.2025.1645290)

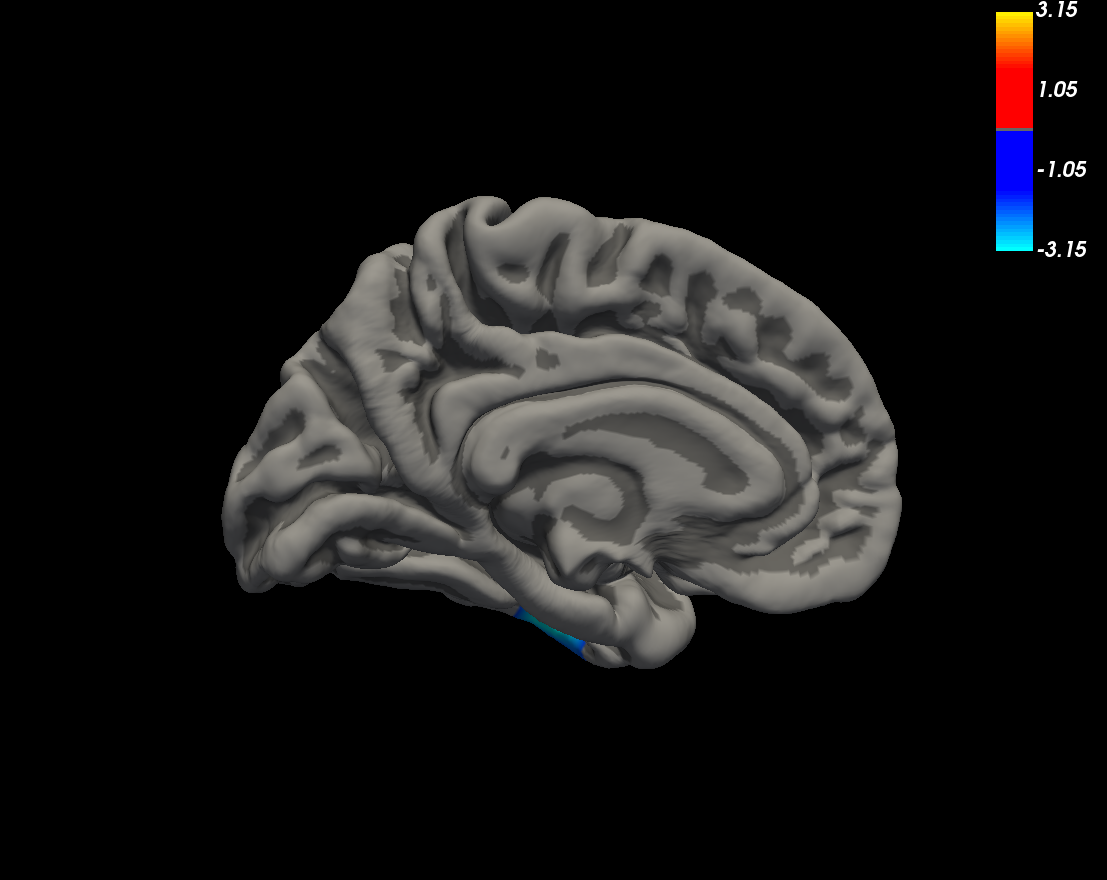

Supplement: Supplementary file 1 [file Data_Sheet_1.ZIP › data/data/cortical thickness/lhEDSvsHC2.png]

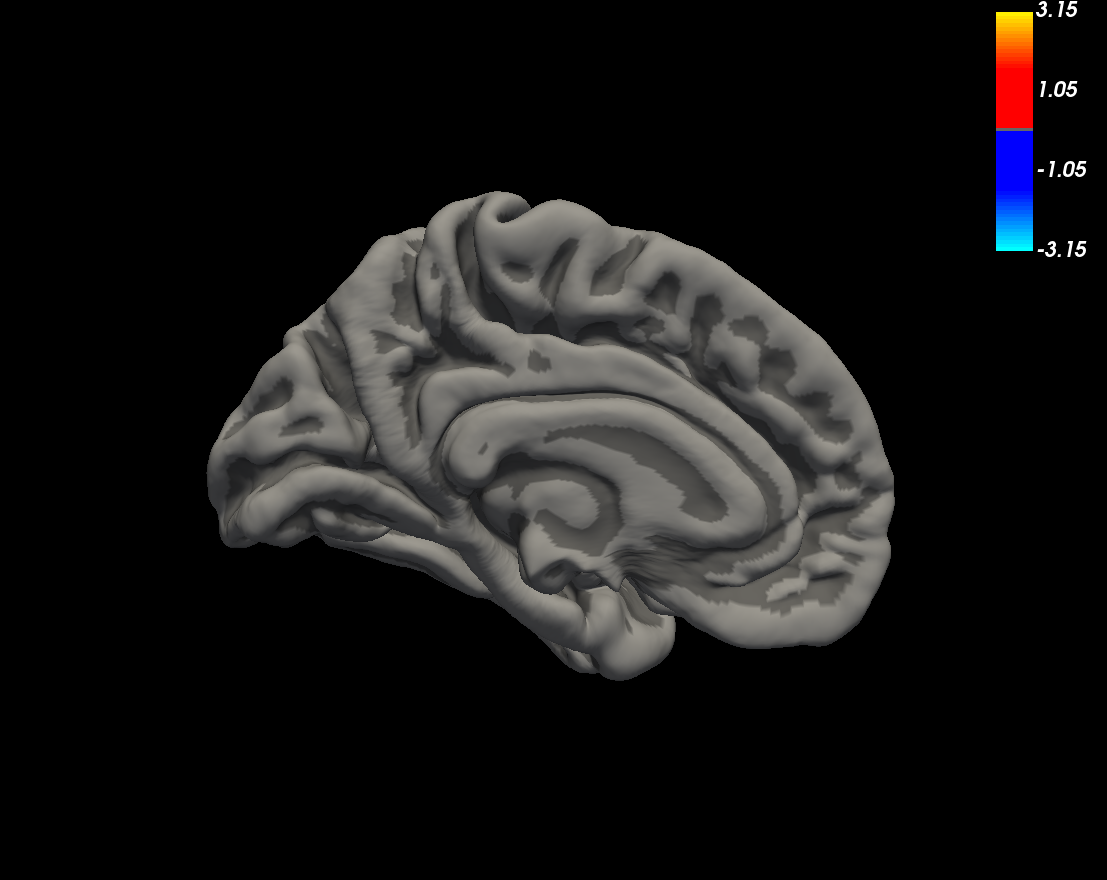

Supplement: Supplementary file 1 [file Data_Sheet_1.ZIP › data/data/cortical thickness/lhEDSvsnEDS.png]

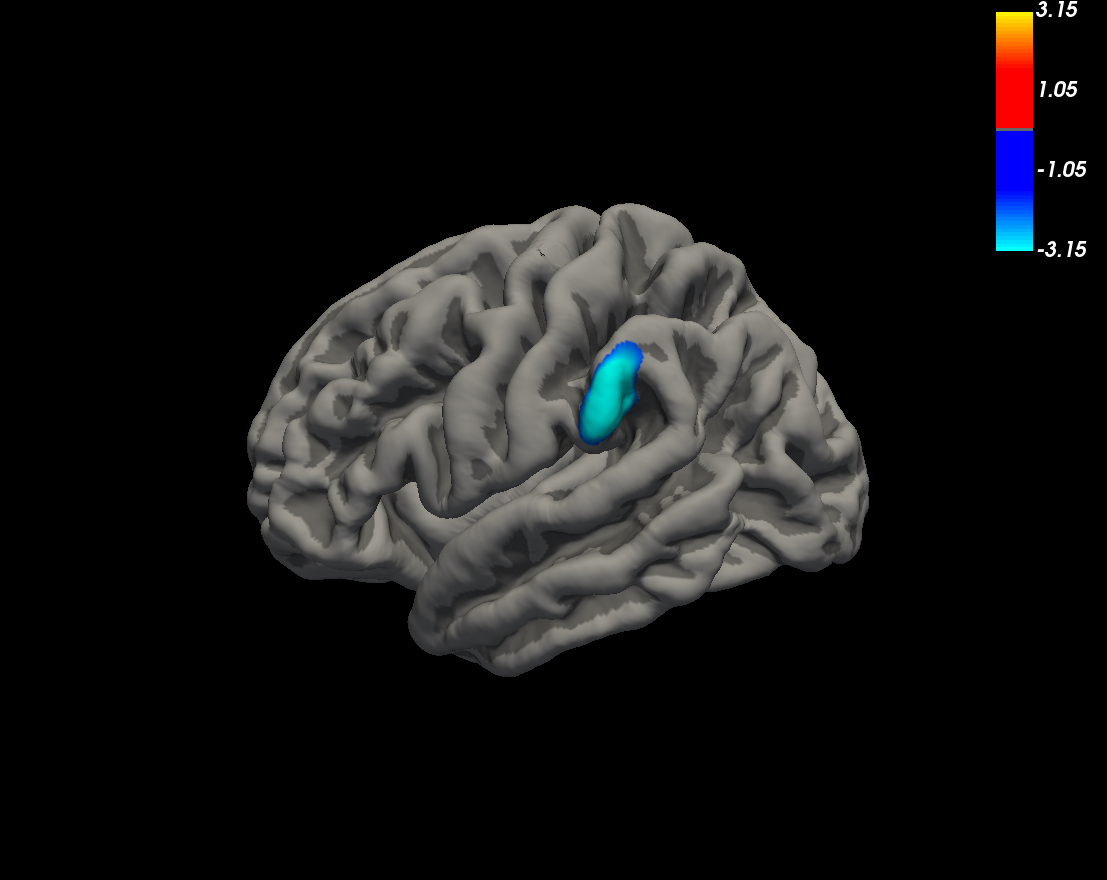

Supplement: Supplementary file 1 [file Data_Sheet_1.ZIP › data/data/cortical thickness/lhEDSvsnEDS2.png]

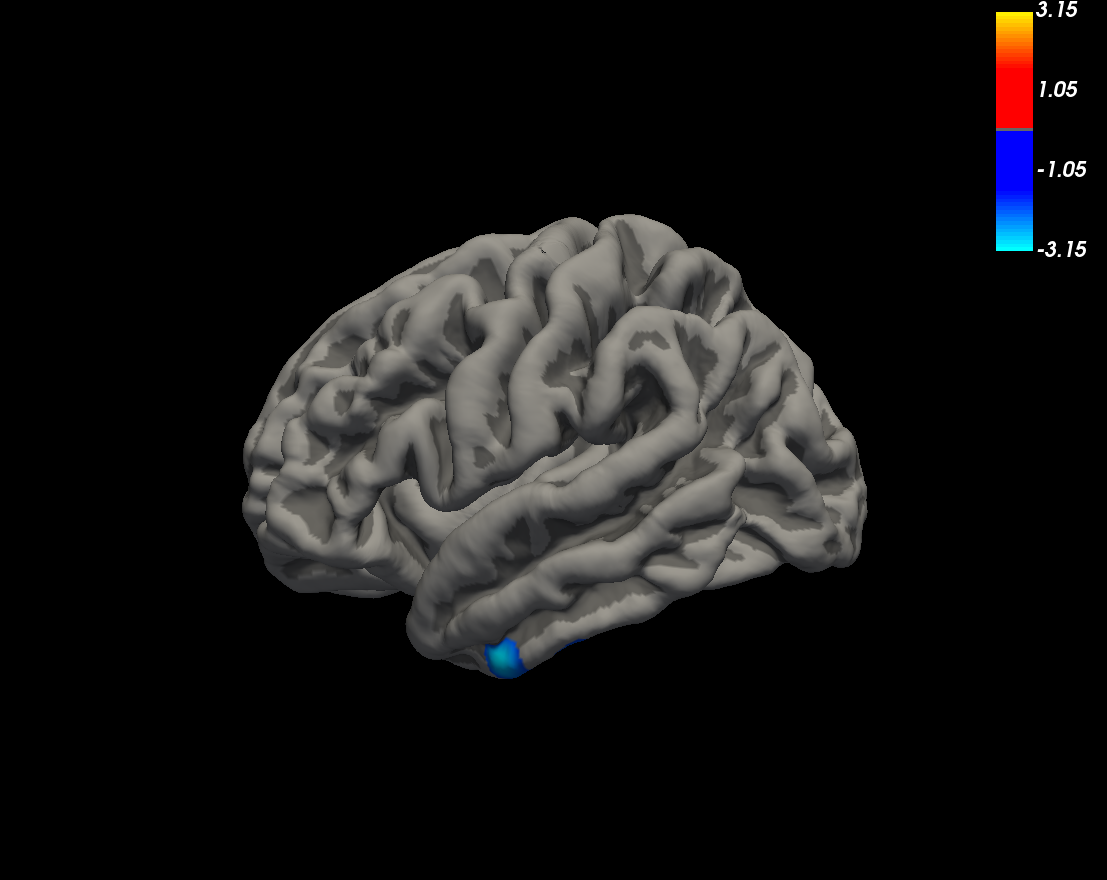

Supplement: Supplementary file 1 [file Data_Sheet_1.ZIP › data/data/cortical thickness/lhEDSvxHC.png]

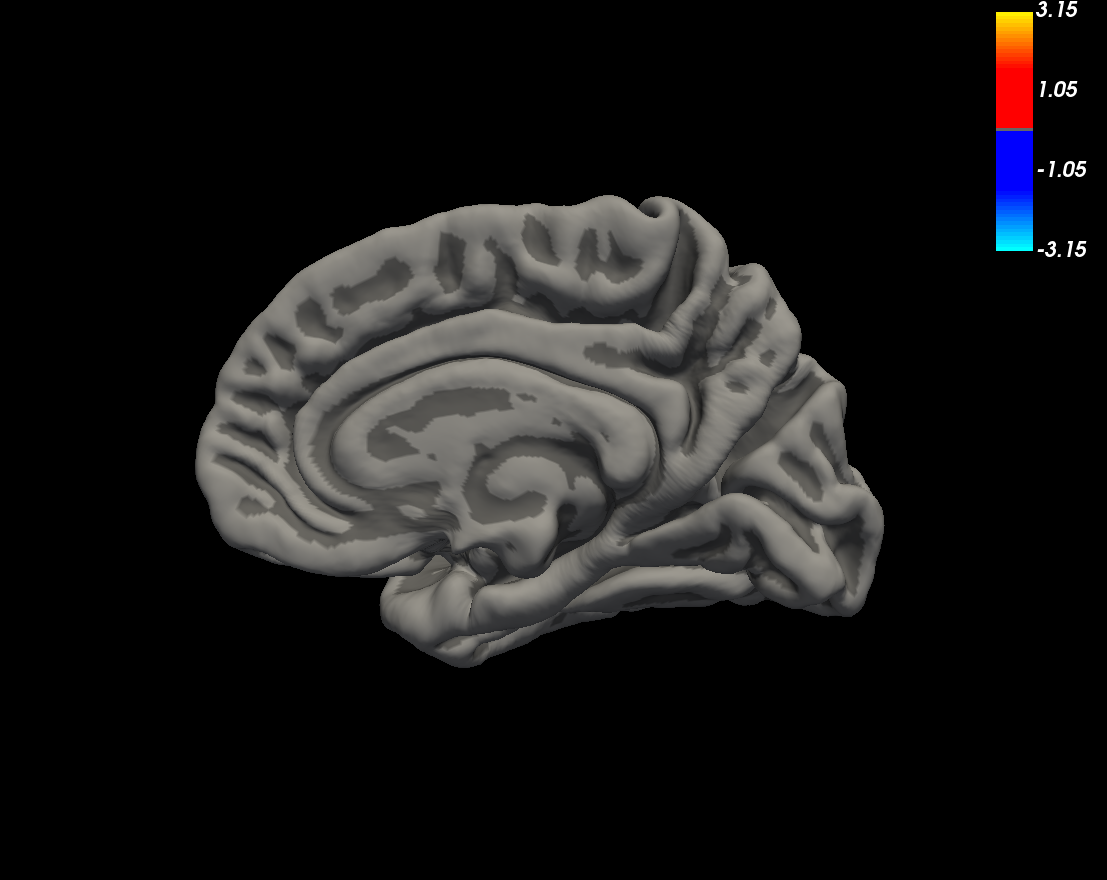

Supplement: Supplementary file 1 [file Data_Sheet_1.ZIP › data/data/cortical thickness/rhEDSvsHC.png]

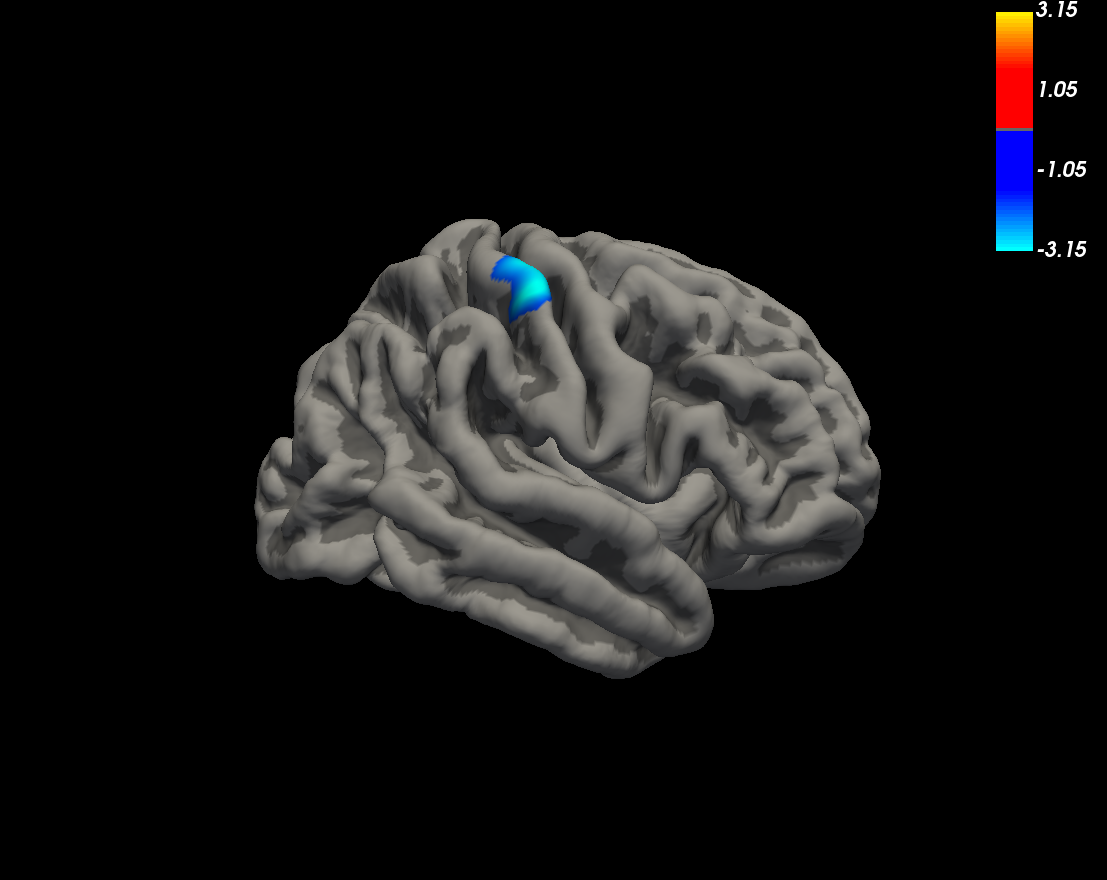

Supplement: Supplementary file 1 [file Data_Sheet_1.ZIP › data/data/cortical thickness/rhEDSvsHC2.png]

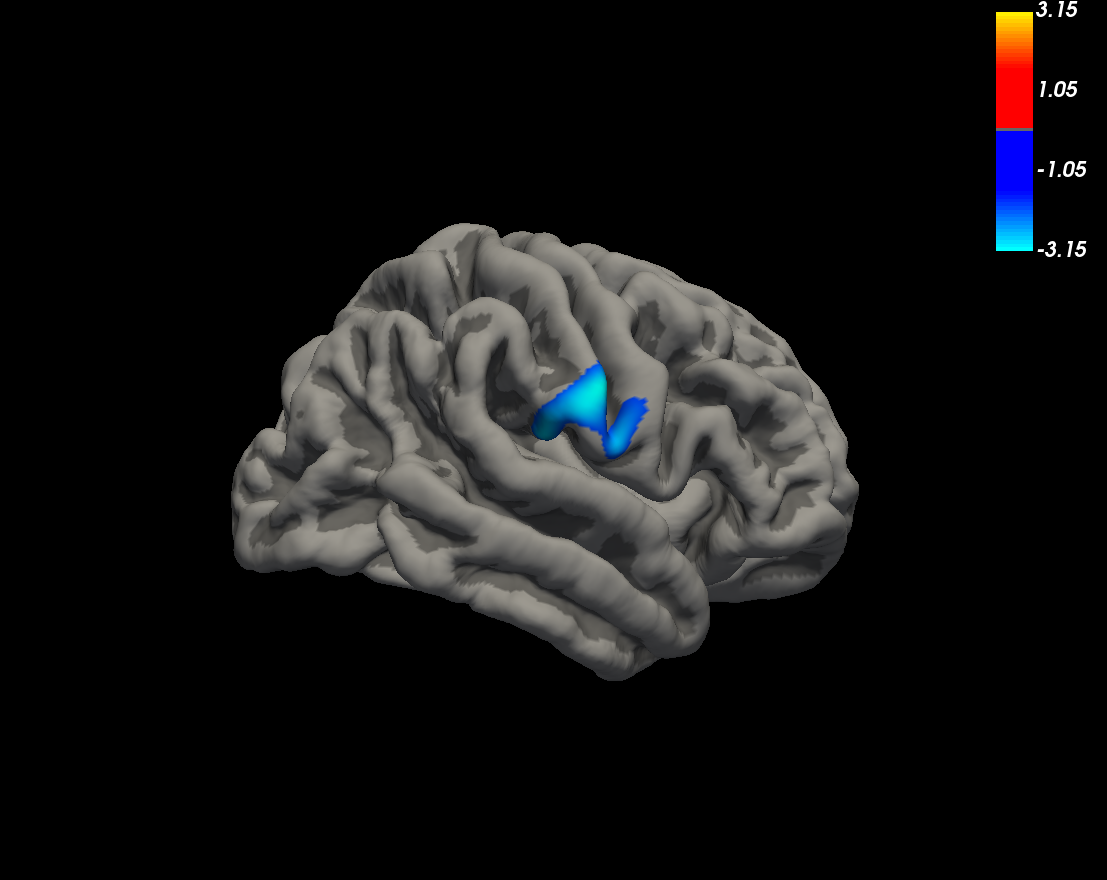

Supplement: Supplementary file 1 [file Data_Sheet_1.ZIP › data/data/cortical thickness/rhEDSvsnEDS.png]

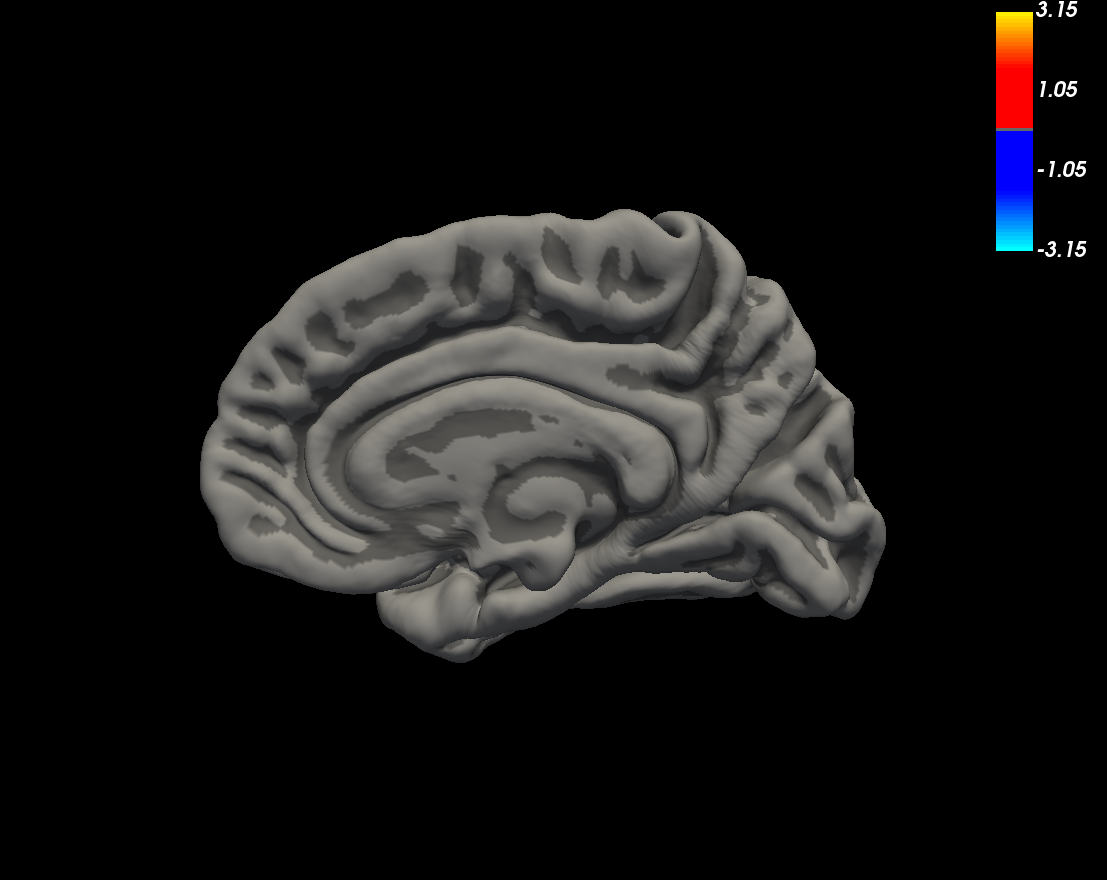

Supplement: Supplementary file 1 [file Data_Sheet_1.ZIP › data/data/cortical thickness/rhEDSvsnEDS2.png]

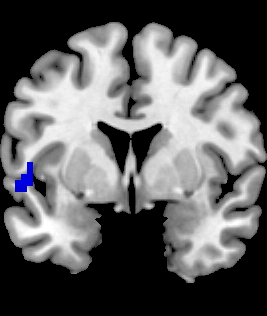

Supplement: Supplementary file 1 [file Data_Sheet_1.ZIP › data/data/FC/Postcentral_R_Frontal_Inf_Oper_L/111.png]

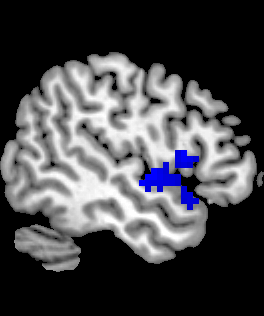

Supplement: Supplementary file 1 [file Data_Sheet_1.ZIP › data/data/FC/Postcentral_R_Frontal_Inf_Oper_L/22222.png]

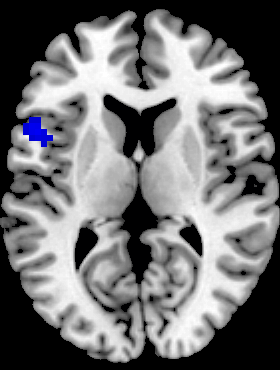

Supplement: Supplementary file 1 [file Data_Sheet_1.ZIP › data/data/FC/Postcentral_R_Frontal_Inf_Oper_L/3333.png]

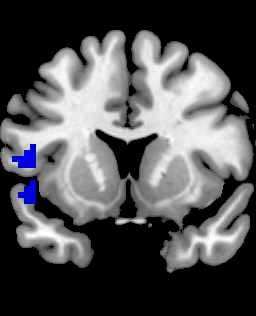

Supplement: Supplementary file 1 [file Data_Sheet_1.ZIP › data/data/FC/Postcentral_R_Paracentral_Lobule_L/4444.png]

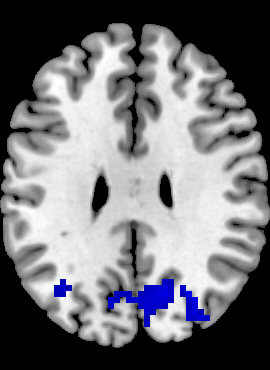

Supplement: Supplementary file 1 [file Data_Sheet_1.ZIP › data/data/FC/Postcentral_R_Temporal_Mid_L/2222.png]

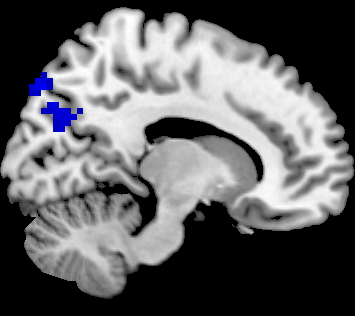

Supplement: Supplementary file 1 [file Data_Sheet_1.ZIP › data/data/FC/Postcentral_R_Temporal_Mid_L/2222222.png]

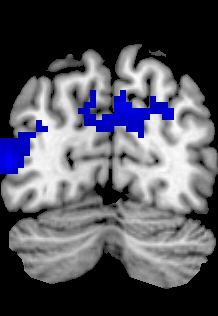

Supplement: Supplementary file 1 [file Data_Sheet_1.ZIP › data/data/FC/Postcentral_R_Temporal_Mid_L/444.png]

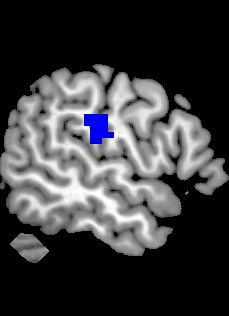

Supplement: Supplementary file 1 [file Data_Sheet_1.ZIP › data/data/FC/SupraMarginal_L_Postcentral_L/1111.png]

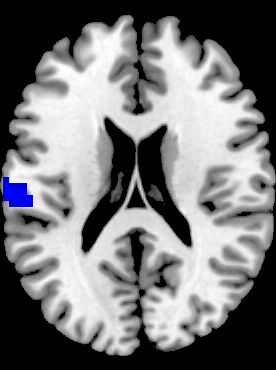

Supplement: Supplementary file 1 [file Data_Sheet_1.ZIP › data/data/FC/SupraMarginal_L_Postcentral_L/222.png]

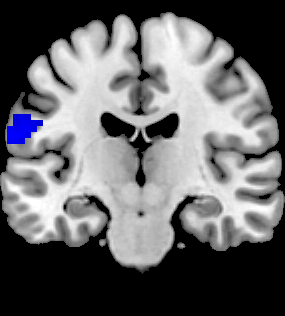

Supplement: Supplementary file 1 [file Data_Sheet_1.ZIP › data/data/FC/SupraMarginal_L_Postcentral_L/333.png]
